# Mobilization-Based Exercise Frequency and Dynamic Postural Control in Orchestra Musicians: An Exploratory Controlled Intervention Study

**DOI:** 10.3390/healthcare14183006

**Published:** 2026-09-14

**Authors:** Enes Furkan Danacı, Özgür Eken, Hanifi Korkmaz, Muhammed Furkan Arpacı, Monira I. Aldhahi

**Affiliations:** 1 Music Education Program, Department of Fine Arts Education, Faculty of Education, İnönü University, 44280 Malatya, Türkiye; enesfurkan.danaci@inonu.edu.tr; 2 Department of Physical Education and Sport Teaching, Faculty of Sport Sciences, İnönü University, 44280 Malatya, Türkiye; ozgur.eken@inonu.edu.tr; 3 Medical Health Services and Vocational School, Malatya Turgut Özal University, 44210 Malatya, Türkiye; hanifi.korkmaz@ozal.edu.tr; 4 Department of Anatomy, Basic Medical Sciences, Faculty of Medicine, Malatya Turgut Özal University, 44210 Malatya, Türkiye; furkan.arpaci@ozal.edu.tr; 5 Department of Rehabilitation Sciences, College of Health and Rehabilitation Sciences, Princess Nourah bint Abdulrahman University, P.O. Box 84428, Riyadh 11671, Saudi Arabia

**Keywords:** posturography, limits of stability, reaction time, performing arts medicine, sensorimotor function, exercise dose

## Abstract

**Highlights:**

**What are the main findings?**

- Twice-weekly mobilization-based exercise was associated with a greater reduction in Limits of Stability Reaction Time than no intervention.
- Static sensory-dependent CTSIB outcomes did not show intervention-specific effects and appeared susceptible to repeated-testing or familiarization effects.

**What are the implications of the main findings?**

- Mobilization may be more relevant to dynamic movement initiation than to quiet-standing sensory integration in orchestra musicians.
- Future rehabilitation trials in musicians should use larger samples, prospective registration with a predefined primary outcome, documented and concealed randomization, familiarization sessions, blinded assessments, and active or sham comparators.

**Abstract:**

Background/Objectives: Orchestra musicians are exposed to prolonged asymmetric postures and repetitive upper-quarter loading that may influence postural-control strategies. This exploratory controlled intervention study examined whether a four-week mobilization-based exercise program performed once or twice weekly was associated with changes in static sensory-dependent balance and dynamic voluntary weight-shifting control. Methods: Twenty-three university orchestra musicians (5 males, 18 females; mean age 24.57 ± 3.41 years) were allocated to a no-intervention control group (*n* = 7), a once-weekly mobilization group (EG1; *n* = 8), or a twice-weekly mobilization group (EG2; *n* = 8). Because no contemporaneous documentation of the allocation-sequence generation could be retrieved, the study is reported as a non-randomized exploratory controlled intervention study, and it was registered retrospectively at ClinicalTrials.gov (NCT07632950; first posted 8 June 2026, after study completion). Postural control was assessed at baseline, Week 1, and Week 4 using CTSIB and LOS tests. Results: No between-group differences were detected for any CTSIB outcome at any time point or for change scores. Although selected CTSIB variables changed within the control and EG2 groups, the absence of between-group differences indicates that these changes were not intervention-specific. For LOS outcomes, change-score comparisons were significant for Reaction Time (H = 9.076, *p* = 0.011, ε^2^ = 0.354) and Directional Control (H = 6.214, *p* = 0.045, ε^2^ = 0.211). After Bonferroni adjustment, only the greater reduction in Reaction Time in EG2 versus control remained significant (*p_adj* = 0.027). Conclusions: In this small exploratory study, twice-weekly mobilization was associated with faster LOS movement initiation, but the absolute and clinical importance of this change remains uncertain. CTSIB outcomes did not demonstrate an intervention-specific effect; therefore, the findings should be considered hypothesis-generating.

## 1. Introduction

Postural control integrates visual, vestibular, somatosensory, and proprioceptive information to maintain or restore the body’s center of mass within the base of support [1,2,3]. Static and dynamic tests capture different components of this process: the Clinical Test we of Sensory Interaction on Balance (CTSIB) evaluates quiet-standing stability under altered sensory conditions, whereas the Limits of Stability (LOS) test evaluates the initiation, direction, and control of voluntary center-of-gravity displacement [4,5]. Consequently, changes in one domain should not be assumed to generalize to the other [6,7,8,9,10].

Orchestra musicians are exposed to prolonged sitting or standing, asymmetric upper-limb positions, sustained cervical loading, and repetitive fine-motor activity. These demands are associated with frequent neck, shoulder, upper-back, and lumbar complaints [11,12,13,14,15,16,17,18]. Although musician-health research has extensively described pain, ergonomic risk, and self-reported limitations, objective posturographic intervention evidence remains limited [19,20,21,22].

Exercise, education, manual therapy, and multimodal prevention strategies have been proposed for musicians, but the evidence is constrained by small samples, heterogeneous protocols, and limited use of objective balance outcomes [23,24,25,26,27,28,29,30,31,32]. Mobilization of the cervical, thoracic, scapulothoracic, pectoral, and shoulder regions may influence movement readiness and proprioceptive input during goal-directed postural tasks [33,34,35,36,37]. However, mobilization without progressive balance, strengthening, perturbation, or task-specific sensorimotor training may be less likely to alter static sensory integration [34,35,36,37].

Repeated posturography can also produce practice or familiarization effects, particularly in tasks involving altered sensory conditions or visual feedback [4,5,7,8]. In short trials, such changes may be mistaken for intervention effects unless between-group patterns and control-group responses are considered.

Therefore, this exploratory controlled intervention study examined whether once- and twice-weekly mobilization-based exercise produced different changes in static sensory-dependent balance and dynamic voluntary weight-shifting control over four weeks. LOS Reaction Time was designated as the primary outcome during the analysis stage rather than in an a priori protocol or statistical analysis plan; all other LOS and CTSIB variables were treated as secondary and exploratory outcomes. We hypothesized that twice-weekly mobilization would produce a greater reduction in LOS Reaction Time than once-weekly mobilization or no intervention, whereas CTSIB changes would be smaller and less intervention-specific.

## 2. Materials and Methods

### 2.1. Study Design and Setting

This exploratory controlled intervention study used a three-group repeated-measures design to examine the effects of mobilization-based exercise frequency on postural control in university orchestra musicians. Participants were allocated to a no-intervention control group, a once-weekly mobilization group (EG1), or a twice-weekly mobilization group (EG2). Assessments were conducted at baseline, Week 1, and Week 4. The study was considered exploratory because the achieved sample size was below the a priori target, because the method of allocation-sequence generation could not be documented from contemporaneous records, and because allocation concealment, assessor blinding, and an active or sham control condition were not implemented. Reporting was structured using those items of the CONSORT 2025 statement and its explanation and elaboration guidance that are applicable to a small exploratory controlled intervention study (Table S1); because random-sequence generation could not be documented, the study is not presented as a randomized trial [38,39]. The study was registered retrospectively at ClinicalTrials.gov (NCT07632950; first submitted 2 June 2026, first posted 8 June 2026). Enrolment began on 5 March 2026, and the study was completed on 5 May 2026, so the record was submitted approximately four weeks after the study had ended, and no prospective protocol or statistical analysis plan was registered or archived before the data were examined. Because the registry entry was itself completed retrospectively, it retains the wording used at the time of registration: it records the allocation as randomized and lists the interim assessment as Week 2. Neither description is adopted here. The allocation procedure cannot be evidenced (Section 2.3), and the interim measurement was performed one week after the start of the intervention; the present report therefore describes the study as a non-randomized exploratory controlled intervention study and reports the interim measurement as the Week 1 assessment.

### 2.2. Participants

The sample consisted of 23 musicians (5 males, 18 females; age: 24.57 ± 3.41 years; range: 20–34 years) enrolled in orchestral and chamber music courses at İnönü University. Group sizes were Control *n* = 7, EG1 *n* = 8, and EG2 *n* = 8. Age did not differ significantly among groups (Kruskal–Wallis H = 3.623, *p* = 0.163), and sex distribution was also not significantly different (χ^2^ = 2.638, *p* = 0.267), although expected cell counts were small. Eligibility required active participation in orchestra activities and the ability to attend all assessment and exercise sessions. Participants with neurological, orthopedic, vestibular, systemic, or other health conditions likely to affect balance were excluded. Written informed consent was obtained from all participants. The study was approved by the Health Sciences Scientific Research Ethics Committee of İnönü University (Date: 16 December 2024; Session: 17; Decision No: 2024/9011).

### 2.3. Group Allocation

**Figure 1:**
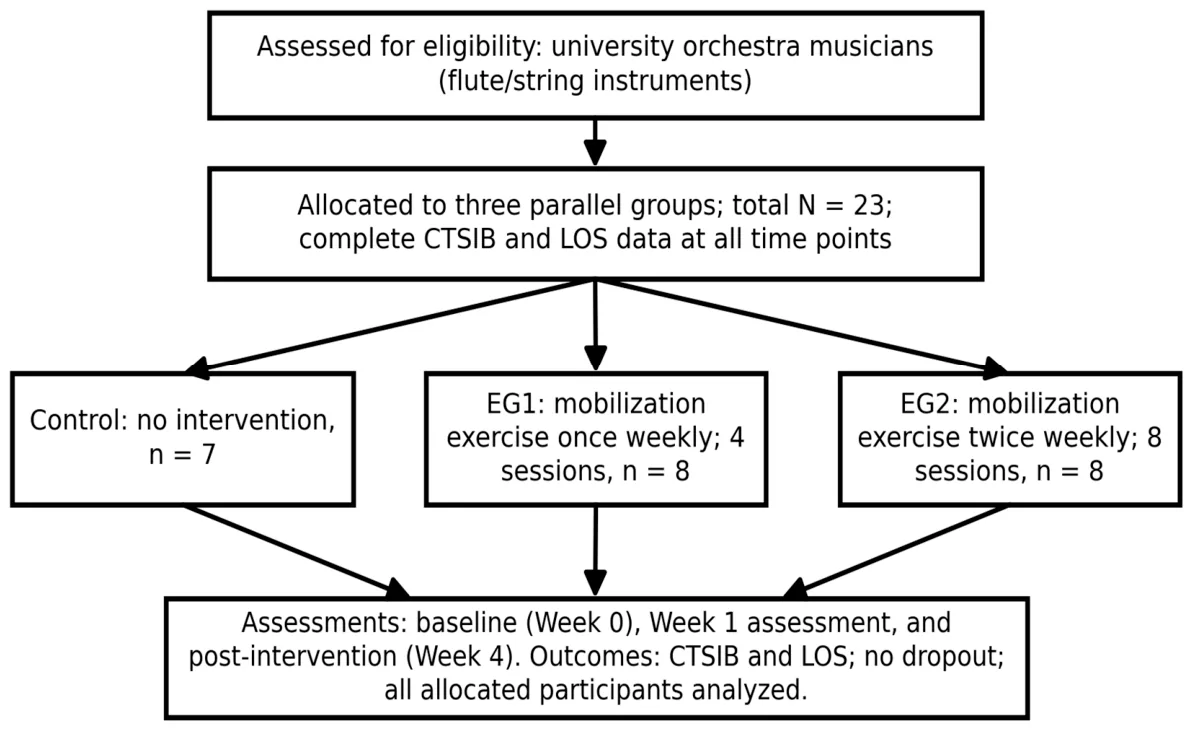
Participant flow diagram showing allocation, measurement time points, and sample sizes. CTSIB = Clinical Test of Sensory Interaction on Balance; LOS = Limits of Stability; EG1 = once-weekly mobilization; EG2 = twice-weekly mobilization.

Participants were assigned to the control, EG1, or EG2 groups in an approximately 1:1:1 ratio (Figure 1). Group assignment was intended to be random; however, no contemporaneous documentary evidence of the allocation procedure is available. The study records that could be retrieved do not identify the random-sequence generation method, any restriction such as blocking or stratification, or the personnel responsible for sequence generation and implementation, and no allocation list, randomization log, or protocol predating enrolment could be located. Because a genuinely random allocation procedure cannot be evidenced, we do not designate the study as a randomized controlled trial and instead describe it throughout as an exploratory controlled intervention study. Allocation concealment and assessor blinding were not implemented. The final group sizes were Control *n* = 7, EG1 *n* = 8, and EG2 *n* = 8. The undocumented allocation procedure and the absence of concealment increase the risk of selection, performance, and detection bias; accordingly, the findings are interpreted as exploratory and hypothesis-generating.

### 2.4. Sample Size and Power

An a priori G*Power calculation (G*Power, version 3.1.9.7; Heinrich-Heine-Universität Düsseldorf, Düsseldorf, Germany) [40] indicated that approximately 25 participants per group would be required to detect a medium effect (f = 0.25) with 80% power at α = 0.05 in a three-group comparison. Because no pilot data or directly comparable trial using StaticVR LOS Reaction Time in orchestra musicians was available when the study was planned, f = 0.25 was selected as a conventional medium standardized effect for the planning calculation rather than as an empirically derived estimate. The target sample could not be achieved because the accessible population of eligible university orchestra musicians was limited. Consequently, the study was underpowered for definitive efficacy testing, and the analysis emphasizes interpretation of effect sizes, consistency of outcome patterns, and cautious inference rather than confirmatory claims.

### 2.5. Outcome Measures

Postural control was assessed with a virtual reality-based StaticVR posturography system (Virtualis, Montpellier, France). LOS Reaction Time, which reflects the speed of initiating voluntary center-of-gravity displacement, was designated as the primary outcome. This designation was not prespecified. No protocol or statistical analysis plan naming a single primary posturographic variable was established before the results were examined, and the retrospective ClinicalTrials.gov record specifies only a generic primary outcome of “change in postural control” measured with the StaticVR system. Reaction Time was designated as the primary outcome during preparation of the statistical analysis, after data collection had been completed and the data had been inspected. The primary-outcome label should therefore be read as an organizing convention for this report rather than as a confirmatory, pre-registered hypothesis test, and all variables are reported with the same exploratory status. Secondary outcomes were CTSIB Composite, Somatosensory, Visual, Vestibular, and Visual Preference scores and LOS Mean Velocity, End Point Excursion, Maximum Excursion, and Directional Control. Higher CTSIB scores indicate better sensory-dependent postural stability. For LOS variables, lower Reaction Time indicates faster movement initiation, whereas higher values for the remaining variables generally indicate better dynamic control, greater stability-limit use, or more accurate directional movement.

### 2.6. Intervention

The four-week mobilization-based program targeted upper-quarter regions exposed to instrument-related static and asymmetric load. The intervention was described in accordance with the Template for Intervention Description and Replication (TIDieR) framework [41]. Each supervised session included Cat-Camel Mobilization (2 × 15 repetitions), Foam Roller Thoracic Extension (2 × 15 repetitions), Scapular Retraction/Protraction (2 × 15 repetitions), Pectoralis Minor Wall Stretch (2 × 30 s), Cervical Side Glide (2 × 15 repetitions), and Shoulder Controlled Articular Rotations (2 × 12 repetitions). Exercises were performed with controlled movement through a comfortable, pain-free range while the supervising researcher monitored technique and alignment. Approximately 30–60 s was allowed between sets or exercises for repositioning and recovery. No external load or target rating of perceived exertion was prescribed. The set, repetition, and hold-time volume remained fixed throughout the four weeks; exercise frequency, rather than within-session progression, constituted the planned dose contrast. Sessions were stopped if pain, dizziness, excessive discomfort, or another safety concern occurred. Each session lasted approximately 20–25 min. EG1 completed one supervised session per week for four weeks (4 sessions), whereas EG2 completed two supervised sessions per week for four weeks (8 sessions). Attendance was recorded, adherence was 100% in both exercise groups, and no adverse events were reported.

### 2.7. Statistical Analysis

Participant characteristics are reported as mean ± SD, and outcome variables as median (minimum-maximum) because of the small group sizes and distributional characteristics. Shapiro–Wilk tests identified non-normality in 17 of 90 group-by-time distributions (18.9%); therefore, non-parametric analyses were used. Between-group comparisons at baseline, Week 1, Week 4, and for posttest–pretest change scores used Kruskal–Wallis H tests, followed when significant by Bonferroni-adjusted Mann–Whitney U tests. Within-group changes across the three time points used Friedman tests with Kendall’s W, followed when significant by Bonferroni-adjusted Wilcoxon signed-rank tests. Kruskal–Wallis epsilon-squared (ε^2^) quantified between-group effects; negative estimates were truncated to zero. Statistical significance was set at *p* < 0.05 and adjusted post hoc significance at *p_adj* < 0.05. Mean ± SEM was used only for figures. Given the small exploratory sample and multiple secondary outcomes, findings were interpreted cautiously as hypothesis-generating. Analyses were performed in Python 3.11 (Python Software Foundation, Wilmington, DE, USA) using SciPy-based procedures [42].

## 3. Results

All participants completed all three measurement sessions and were included in the final analysis. Table 1 summarizes baseline participant characteristics. The groups did not differ significantly in age or sex distribution; however, the study was not powered to confirm demographic equivalence, and the unequal sex distribution should be considered when interpreting generalizability.

### 3.1. CTSIB Outcomes

CTSIB descriptive values are presented in Table 2. Baseline CTSIB scores were broadly similar across groups. Somatosensory and Visual Preference scores were close to ceiling in all groups, restricting sensitivity for further improvement. Visual and Vestibular scores increased from baseline to Week 4 in the control group and EG2, while EG1 showed smaller changes in the same direction. Because no between-group change-score comparison was significant, none of these within-group patterns can be attributed specifically to the intervention.

Table 2 shows stable Composite scores and ceiling effects for the Somatosensory and Visual Preference components, indicating restricted sensitivity for detecting intervention-related change in static sensory-dependent balance.

Table 3 presents CTSIB inferential statistics. No Kruskal–Wallis comparison was significant for any CTSIB variable at baseline, Week 1, Week 4, or for posttest–pretest change scores. Thus, the CTSIB data do not support a differential intervention effect. Significant within-group changes in selected variables occurred in more than one group, including the no-intervention control group. These findings may reflect repeated-test exposure, day-to-day variability, unmeasured physical-activity or fitness differences, regression to the mean, or other uncontrolled influences; the present design cannot distinguish among these explanations.

**Figure 2:**
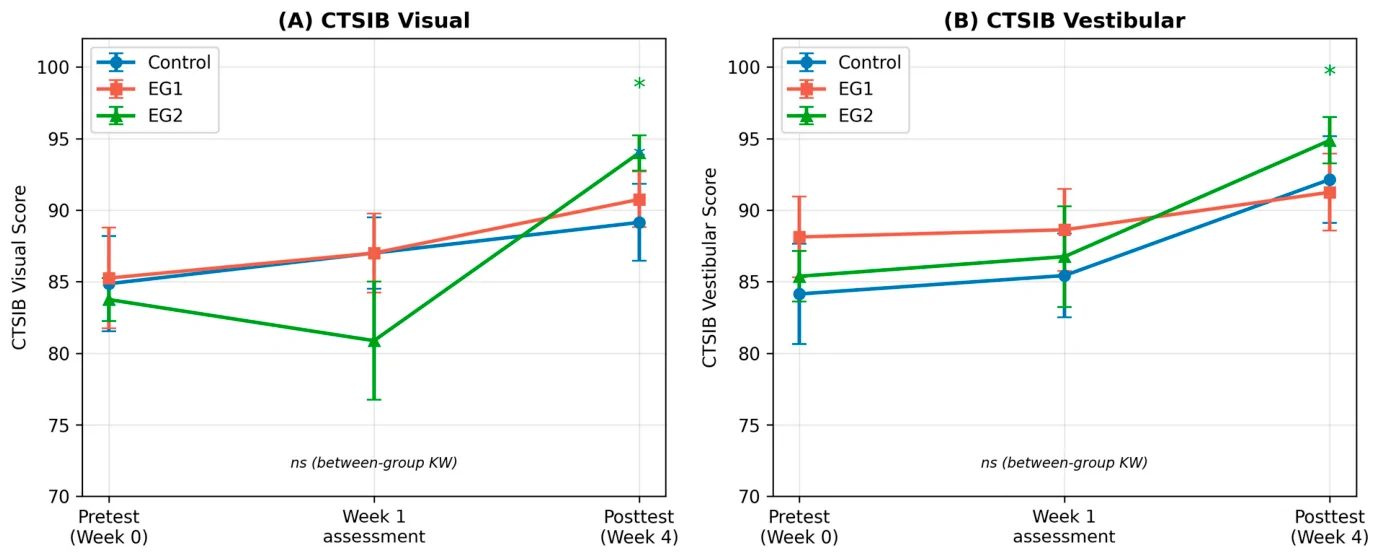
CTSIB Visual (**A**) and Vestibular (**B**) scores across time by group. Values are mean ± SEM for visualization. Asterisks denote significant Bonferroni-adjusted within-group baseline-to-Week 4 comparisons. Because between-group tests were non-significant, these patterns should be interpreted cautiously. These figures are descriptive visualizations only and should not be interpreted as evidence of between-group efficacy because the corresponding group comparisons were non-significant.

No CTSIB between-group comparison was significant (Figure 2). The within-group Visual and Vestibular changes observed in EG2 were accompanied by changes in the control group and smaller changes in EG1; therefore, they should be treated as non-specific temporal patterns rather than evidence of efficacy.

**Figure 3:**
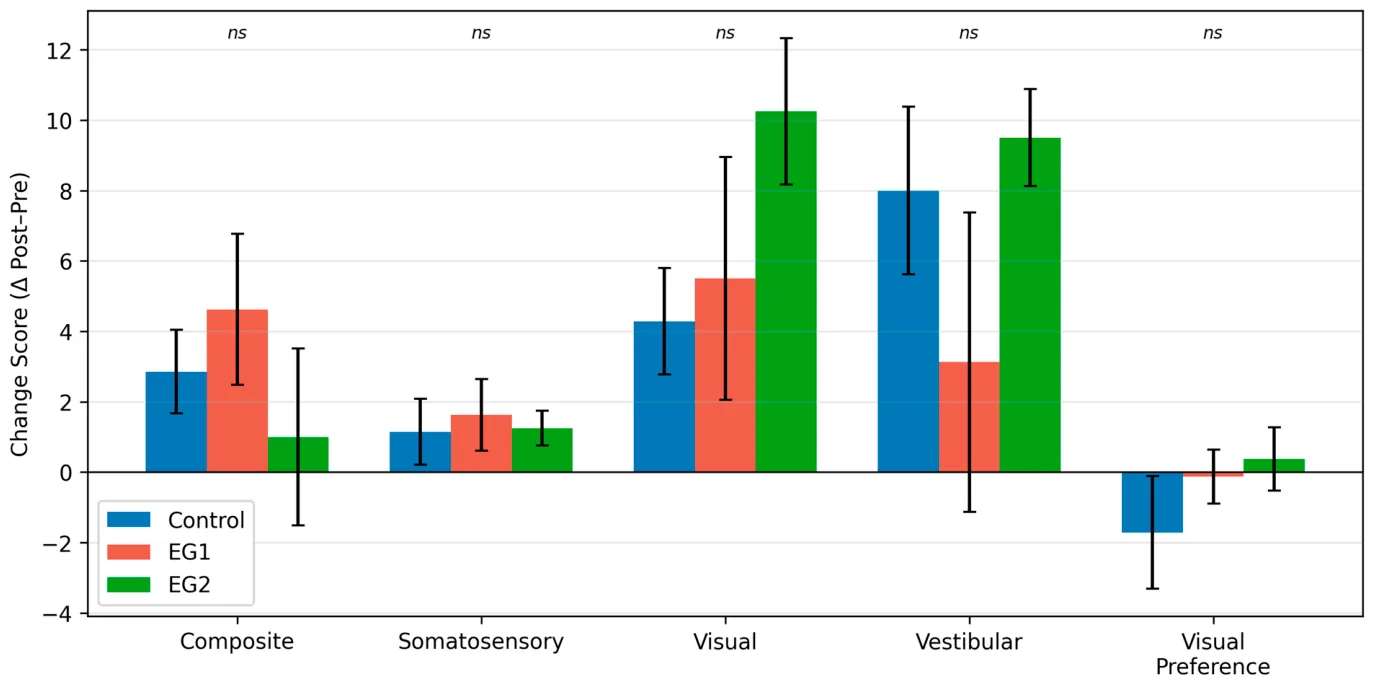
CTSIB posttest–pretest change scores by group. Values are mean ± SEM. These figures are descriptive visualizations only and should not be interpreted as evidence of between-group efficacy because the corresponding group comparisons were non-significant.

All Kruskal–Wallis between-group comparisons were non-significant, indicating no differential CTSIB response among groups. The visual pattern suggests that EG2 tended to show larger Visual and Vestibular gains, but these changes did not exceed the variability and repeated-testing effects observed across groups (Figure 3).

### 3.2. LOS Outcomes

LOS descriptive values are presented in Table 4. EG2 had a slower baseline Reaction Time than the other groups and subsequently decreased from 1.15 s at baseline to 0.77 s at Week 4. EG1 showed a smaller decrease, whereas the control group showed little change. Because EG2 began from a poorer value, regression to the mean and baseline imbalance may have contributed to the magnitude of its observed improvement. Directional Control increased most clearly in EG2, while Mean Velocity, End Point Excursion, and Maximum Excursion did not show a consistent intervention-specific pattern.

For Reaction Time, lower values indicate better performance. For Mean Velocity, End Point Excursion, Maximum Excursion, and Directional Control, higher values indicate better performance. The clearest descriptive pattern was observed in EG2, which improved from 1.15 to 0.77 s in Reaction Time and from 72.0 to 83.5% in Directional Control, whereas End Point Excursion and Maximum Excursion did not show a consistent intervention-specific pattern.

**Figure 4:**
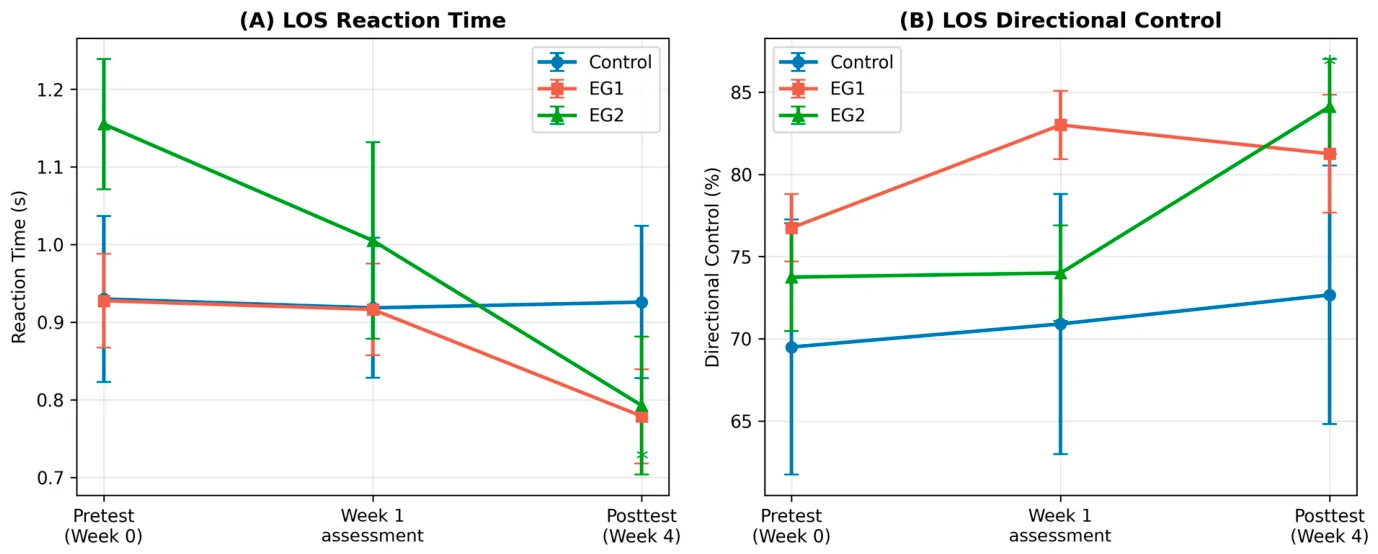
LOS Reaction Time (**A**) and Directional Control (**B**) across measurement time points by group.

**Figure 5:**
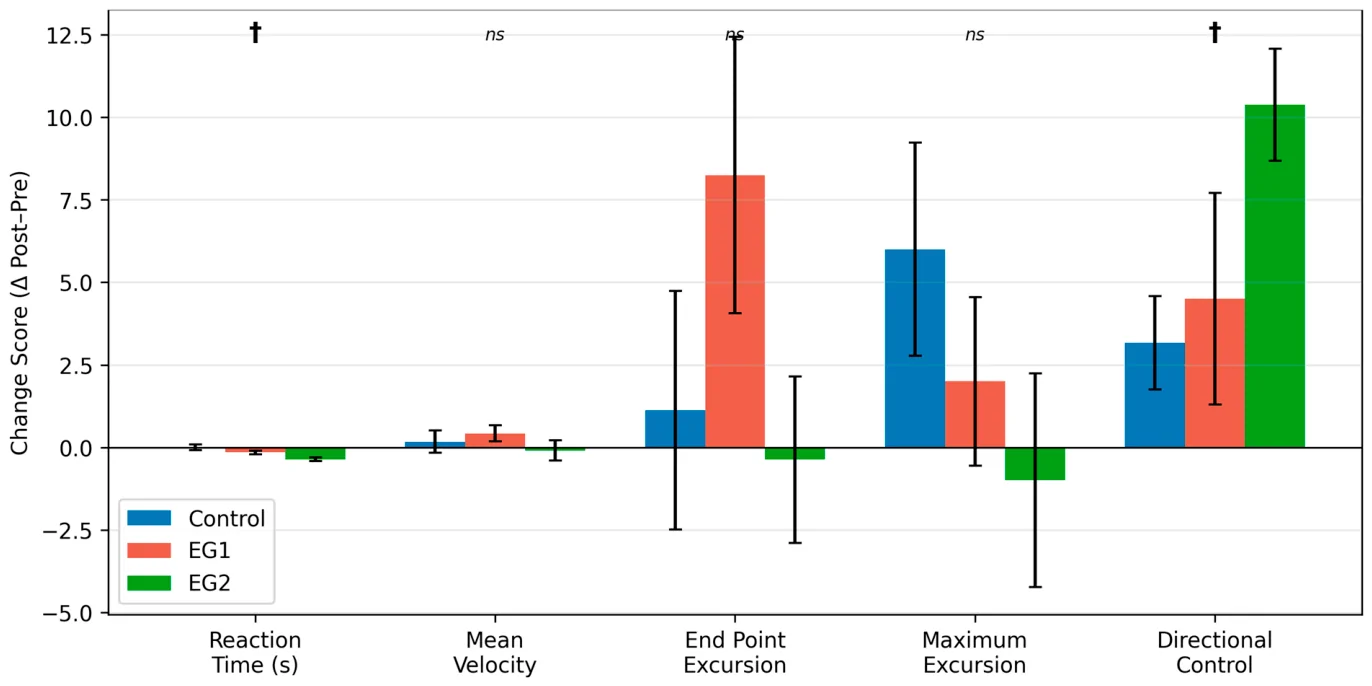
LOS posttest–pretest change scores by group. Values are mean ± SEM. † indicates significant Kruskal–Wallis between-group change-score comparison.

Table 5 presents LOS inferential statistics. Between-group change-score comparisons were significant for Reaction Time and Directional Control only. Reaction Time showed a moderate-to-large between-group effect (H = 9.076, *p* = 0.011, ε^2^ = 0.354), with Bonferroni-adjusted post hoc testing indicating a greater reduction in Reaction Time in EG2 than in the control group (*p_adj* = 0.027). Directional Control showed a significant omnibus change-score effect (H = 6.214, *p* = 0.045, ε^2^ = 0.211), although pairwise post hoc comparisons did not remain significant after Bonferroni correction. Therefore, the Directional Control finding should be interpreted as preliminary. EG2 showed large within-group effects for both Reaction Time and Directional Control (W = 0.766 for both), whereas Mean Velocity, End Point Excursion, and Maximum Excursion did not show significant intervention-specific between-group change. The trajectories for LOS Reaction Time and Directional Control are shown in Figure 4, and the LOS posttest–pretest change-score comparisons are shown in Figure 5.

Bonferroni-adjusted pairwise post hoc significance was observed only for Reaction Time change between Control and EG2 (*p_adj* = 0.027). The Directional Control change-score comparison was significant at the omnibus level (H = 6.214, *p* = 0.045, ε^2^ = 0.211), but pairwise contrasts did not remain significant after Bonferroni correction; therefore, this finding should be interpreted as exploratory. The control-group Directional Control *p* = 0.050 was not considered statistically significant under the predefined *p* < 0.05 threshold.

Values are mean ± SEM. EG2 demonstrated large within-group effects for both Reaction Time and Directional Control (W = 0.766 for each). The Reaction Time trajectory shows the clearest preliminary between-group statistical signal because Reaction Time decreased in EG2 while the control group remained essentially unchanged, and the Control-versus-EG2 change contrast remained significant after Bonferroni correction. This descriptive pattern should not be read as evidence of efficacy.

Between-group change-score analyses showed significant effects for Reaction Time (H = 9.076, *p* = 0.011, ε^2^ = 0.354) and Directional Control (H = 6.214, *p* = 0.045, ε^2^ = 0.211), but not for Mean Velocity, End Point Excursion, or Maximum Excursion. This pattern indicates a preliminary between-group statistical signal confined to selected dynamic-balance variables rather than generalized LOS improvement. No experimental group demonstrated a significant Bonferroni-adjusted baseline-to-Week 1 improvement in CTSIB or LOS variables; therefore, the data do not support a robust acute effect after one week of mobilization-based exercise.

## 4. Discussion

This exploratory controlled intervention study examined whether a four-week mobilization-based exercise program performed once or twice weekly was associated with changes in static sensory-dependent balance and dynamic voluntary weight-shifting control in university orchestra musicians. The main finding was that twice-weekly mobilization was associated with a greater reduction in LOS Reaction Time than no intervention, a change that is compatible with faster initiation of voluntary center-of-gravity movement but that cannot be attributed causally to the intervention under the present design. Directional Control also showed a significant omnibus change-score effect, although pairwise comparisons did not remain significant after Bonferroni adjustment. In contrast, CTSIB outcomes did not show intervention-specific effects, and no consistent improvements were observed for LOS Mean Velocity, End Point Excursion, or Maximum Excursion. These findings are compatible with an association between short-term mobilization and selected components of dynamic postural control, particularly movement initiation, but they do not demonstrate a generalized effect across static and dynamic balance domains, and they do not establish efficacy.

The absence of significant between-group effects for CTSIB outcomes is important. Although some within-group improvements were observed, changes also occurred in the control group, and smaller changes were evident in EG1. Repeated exposure to the protocol is one possible explanation, but it is not the only one. Day-to-day biological variability, baseline imbalance, regression to the mean, and unmeasured physical-activity or fitness characteristics may also have influenced the trajectories. Because habitual physical activity, training history, and objective fitness were not assessed, the study cannot determine which mechanism best explains these non-specific CTSIB changes.

The clearest preliminary between-group statistical signal was observed for LOS Reaction Time, which was designated as the primary outcome after data collection rather than prespecified. EG2 showed a greater reduction than the control group after multiplicity adjustment, whereas EG1 showed a smaller change that did not differ significantly from either group. However, EG2 also started with slower Reaction Time, so baseline imbalance and regression to the mean cannot be excluded. Moreover, the absolute difference between the EG1 and EG2 improvements was small in seconds, and the measurement error and minimal clinically important difference for this specific population are unknown. The result should therefore be interpreted as a preliminary between-group statistical signal rather than as evidence of efficacy or of a meaningful dose–response effect.

The observed Reaction Time change might have practical relevance if replicated, because faster initiation of controlled center-of-gravity displacement could support postural adjustments during instrument handling or task transitions. Nevertheless, no minimal clinically important difference has been established for LOS Reaction Time in orchestra musicians, and the study did not evaluate falls, playing performance, pain, or functional participation. Accordingly, the magnitude cannot yet be classified as clinically meaningful.

Directional Control also showed a significant omnibus change-score comparison, but pairwise comparisons did not remain significant after adjustment. This finding should therefore be interpreted cautiously. Directional Control reflects the accuracy of voluntary movement trajectory toward a target and may be influenced by motor planning, proprioceptive feedback, and postural coordination [4,5]. For orchestra musicians, who frequently maintain prolonged asymmetric postures while coordinating precise upper-limb movements, improvements in directional control may be functionally relevant. Nevertheless, the present study did not assess musical performance, pain, fatigue, or injury risk; therefore, the practical relevance of these posturographic changes remains uncertain.

The difference between CTSIB and LOS outcomes supports a task-specific interpretation. Mobilization-based exercise may be more likely to influence active, goal-directed movement tasks than quiet-standing sensory integration. Static balance under altered sensory conditions may require more specific vestibular, visual, somatosensory, perturbation-based, or balance-oriented training than was included in the present intervention [43,44]. Future programs may therefore benefit from combining mobilization with progressive strengthening, proprioceptive training, balance tasks, ergonomic education, and instrument-specific postural retraining [29,30,31,37].

The apparent frequency-related pattern should be interpreted cautiously. EG2 completed eight supervised sessions, and EG1 completed four; however, EG1 did not differ significantly from either group, and EG2 began with poorer Reaction Time. These data therefore do not establish a dose–response relationship. A larger trial using baseline-adjusted modeling, stratified randomization, and an active comparator is required to determine whether exercise frequency independently influences LOS Reaction Time.

This study contributes preliminary objective posturographic evidence to the performing arts medicine literature. Previous musician health research has largely focused on pain prevalence, ergonomic risk, physiotherapy service use, and self-reported outcomes [11,12,13,14,15,16,17,18,26,27,28,29,30,31,32,45]. By separating static sensory-dependent balance from dynamic voluntary weight-shifting control, the present study provides a more nuanced interpretation of postural-control responses to mobilization-based exercise in orchestra musicians. However, the findings should be viewed as hypothesis-generating rather than definitive.

Several limitations should be acknowledged. First, the achieved sample (*n* = 23; 7–8 participants per group) was substantially below the a priori target of approximately 75 participants (25 per group), leaving the study underpowered for definitive efficacy testing. In contrast with the broader musician-intervention literature that commonly evaluates pain, disability, posture, or physical function [23,24,25,26,27,28,29,30,31,32,45], the present study used LOS Reaction Time as its primary posturographic outcome; directly comparable intervention estimates were not identified in the cited literature, preventing a valid quantitative pooled comparison. The observed between-group Reaction Time effect (ε^2^ = 0.354; adjusted Control-versus-EG2 *p* = 0.027) should therefore be treated as an internal estimate requiring replication rather than as an established benchmark. Second, the allocation procedure could not be reconstructed from contemporaneous records, so the study is reported as a non-randomized exploratory controlled intervention study and residual selection bias cannot be excluded; registration at ClinicalTrials.gov was retrospective, and because no protocol or statistical analysis plan predated inspection of the data, the primary-outcome designation was made post hoc and outcome-selection bias cannot be excluded. Allocation concealment and assessor blinding were also not implemented, and the control group received neither a sham nor an active intervention. Third, no pre-baseline familiarization session was used, which may explain control-group CTSIB changes. Fourth, instrument type, weekly practice duration, pain, previous injury, physical activity, sleep, fatigue, and psychological state were not controlled. Fifth, the intervention lasted four weeks, and no follow-up assessed durability. Sixth, cervical proprioception, range of motion, muscle activation, strength, pain, and movement kinematics were not measured. Finally, multiple outcomes and comparisons increased false-positive risk despite post hoc adjustment. These limitations restrict precision, generalizability, causal attribution, and clinical interpretation. The sample combined 5 men and 18 women, and the small cell counts precluded reliable sex-stratified subgroup analysis [46]. Excluding men after group allocation would violate the analysis population defined at allocation and further reduce power; nevertheless, sex imbalance limits generalizability and should be addressed through stratified recruitment in future trials. Habitual physical activity, training history, and objective physical-fitness indicators were not measured, although they may affect vestibular, neuromuscular, and postural-control performance. Participants were recruited from a single university orchestra, which further limits external validity across professional musicians, different instruments, age groups, and cultural settings.

## 5. Conclusions

In this exploratory controlled intervention study, twice-weekly mobilization-based exercise was associated with a greater reduction in LOS Reaction Time than no intervention, but baseline imbalance, small absolute differences, and unknown clinical thresholds limit interpretation. Directional Control showed an omnibus change that was not confirmed in adjusted pairwise comparisons, and CTSIB outcomes did not demonstrate intervention-specific effects. These findings are hypothesis-generating rather than confirmatory. Future adequately powered, sex-balanced, multicenter trials should be prospectively registered with a predefined primary outcome, measure baseline physical fitness, use documented, concealed, and stratified randomization, incorporate familiarization and active-control conditions, and evaluate whether changes in LOS Reaction Time translate into meaningful functional outcomes for musicians.

## Figures and Tables

**Table 1:** Participant characteristics by group.

Group	*n*	Age, Years (Mean ± SD)	Male/Female
Control	7	26.86 ± 4.38	3/4
EG1	8	23.25 ± 2.25	1/7
EG2	8	23.88 ± 2.70	1/7

Note. Age comparison: Kruskal–Wallis H = 3.623, *p* = 0.163. Sex distribution: χ^2^ = 2.638, *p* = 0.267. Expected sex-count cells were small; therefore, sex distribution should be interpreted descriptively rather than as evidence of definitive baseline equivalence.

**Table 2:** Descriptive statistics for CTSIB variables.

Variable	Time	Control (*n* = 7)	EG1 (*n* = 8)	EG2 (*n* = 8)
Composite	Pretest	78.00 (69.00–82.00)	77.00 (58.00–88.00)	78.50 (74.00–80.00)
	Week 1 assessment	79.00 (71.00–81.00)	77.00 (73.00–82.00)	78.50 (67.00–82.00)
	Posttest	80.00 (77.00–82.00)	80.00 (75.00–94.00)	80.50 (62.00–86.00)
Somatosensory	Pretest	100.00 (79.00–100.00)	99.50 (92.00–100.00)	99.00 (96.00–100.00)
	Week 1 assessment	100.00 (85.00–100.00)	99.50 (90.00–100.00)	99.00 (95.00–100.00)
	Posttest	100.00 (85.00–100.00)	100.00 (97.00–100.00)	100.00 (100.00–100.00)
Visual	Pretest	87.00 (70.00–97.00)	84.50 (69.00–100.00)	84.50 (77.00–89.00)
	Week 1 assessment	88.00 (78.00–97.00)	88.00 (74.00–100.00)	82.50 (62.00–97.00)
	Posttest	90.00 (80.00–100.00)	90.50 (84.00–100.00)	93.50 (90.00–100.00)
Vestibular	Pretest	84.00 (69.00–100.00)	86.00 (80.00–100.00)	84.50 (78.00–93.00)
	Week 1 assessment	85.00 (74.00–100.00)	90.50 (73.00–100.00)	90.00 (67.00–97.00)
	Posttest	90.00 (80.00–100.00)	92.00 (79.00–100.00)	95.50 (88.00–100.00)
Visual Preference	Pretest	99.00 (95.00–100.00)	100.00 (95.00–100.00)	100.00 (93.00–100.00)
	Week 1 assessment	99.00 (96.00–100.00)	97.50 (84.00–100.00)	100.00 (97.00–100.00)
	Posttest	99.00 (93.00–100.00)	99.00 (97.00–100.00)	100.00 (97.00–100.00)

Note. Values are medians (minimum-maximum). Higher CTSIB scores indicate better performance. The term “Week 1 assessment” replaces “acute test” because the measurement was performed after one week of exposure rather than immediately after a single session.

**Table 3:** Between-group and within-group analyses for CTSIB variables.

Variable	KW Pre H(p)	KW Week 1 H(p)	KW Post H(p)	KW Δ H(p)	Control Friedman χ^2^(p), W	EG1 Friedman χ^2^(p), W	EG2 Friedman χ^2^(p), W
Composite	0.930 (0.628)	0.391 (0.822)	0.312 (0.856)	0.393 (0.821)	6.741 (0.034), W = 0.481 *	5.871 (0.053), W = 0.367	5.871 (0.053), W = 0.367
Somatosensory	0.128 (0.938)	0.011 (0.994)	2.528 (0.283)	0.686 (0.709)	0.800 (0.670), W = 0.057	3.111 (0.211), W = 0.194	6.870 (0.032), W = 0.429 *
Visual	0.153 (0.927)	1.673 (0.433)	3.094 (0.213)	3.060 (0.217)	11.565 (0.003), W = 0.826 *	2.774 (0.250), W = 0.173	9.250 (0.010), W = 0.578 *
Vestibular	0.556 (0.757)	0.846 (0.655)	0.894 (0.640)	2.114 (0.347)	11.143 (0.004), W = 0.796 *	3.185 (0.203), W = 0.199	10.750 (0.005), W = 0.672 *
Visual Preference	1.049 (0.592)	5.782 (0.056)	4.524 (0.104)	1.100 (0.577)	1.130 (0.568), W = 0.081	2.000 (0.368), W = 0.125	0.400 (0.819), W = 0.025

Note. KW = Kruskal–Wallis; Δ = posttest–pretest. * *p* < 0.05. Kendall’s W is reported as the Friedman effect-size index.

**Table 4:** Descriptive statistics for LOS variables.

Variable	Time	Control (*n* = 7)	EG1 (*n* = 8)	EG2 (*n* = 8)
Reaction Time	Pretest	0.96 (0.38–1.23)	0.93 (0.67–1.20)	1.15 (0.86–1.50)
	Week 1 assessment	0.98 (0.48–1.19)	0.89 (0.72–1.22)	0.95 (0.55–1.72)
	Posttest	1.01 (0.57–1.17)	0.83 (0.51–0.95)	0.77 (0.51–1.29)
Mean Velocity	Pretest	3.58 (2.86–4.50)	3.25 (1.68–4.10)	2.81 (2.30–4.54)
	Week 1 assessment	3.66 (2.45–5.06)	3.28 (1.69–4.03)	3.02 (2.10–5.05)
	Posttest	3.75 (2.03–5.63)	3.80 (2.67–4.43)	2.98 (2.18–4.03)
End Point Excursion	Pretest	53.00 (45.00–86.00)	59.50 (45.00–76.00)	58.00 (47.00–79.00)
	Week 1 assessment	54.00 (46.00–82.00)	60.50 (45.00–79.00)	62.50 (45.00–79.00)
	Posttest	64.00 (48.00–77.00)	70.50 (56.00–82.00)	58.50 (42.00–74.00)
Maximum Excursion	Pretest	85.67 (73.00–99.00)	88.50 (78.00–95.00)	84.50 (81.00–95.00)
	Week 1 assessment	88.67 (74.00–100.00)	88.00 (78.00–99.00)	87.50 (78.00–101.00)
	Posttest	96.00 (75.00–100.00)	88.00 (86.00–98.00)	87.50 (69.00–92.00)
Directional Control	Pretest	73.00 (28.00–88.00)	77.50 (69.00–84.00)	72.00 (63.00–87.00)
	Week 1 assessment	76.00 (29.00–89.00)	83.00 (75.00–91.00)	73.00 (63.00–87.00)
	Posttest	78.00 (30.00–90.00)	83.50 (61.00–90.00)	83.50 (71.00–97.00)

Note. Values are medians (minimum–maximum).

**Table 5:** Between-group and within-group analyses for LOS variables.

Variable	KW Pre H(p), ε^2^	KW Week 1 H(p), ε^2^	KW Post H(p), ε^2^	KW Δ H(p), ε^2^	Control Friedman χ^2^(p), W	EG1 Friedman χ^2^(p), W	EG2 Friedman χ^2^(p), W
Reaction Time	3.862 (0.145), ε^2^ = 0.093	0.181 (0.913), ε^2^ = 0.000	2.074 (0.354), ε^2^ = 0.004	9.076 (0.011), ε^2^ = 0.354 *	2.000 (0.368), W = 0.143	7.548 (0.023), W = 0.472 *	12.250 (0.002), W = 0.766 *
Mean Velocity	1.841 (0.398), ε^2^ = 0.000	1.011 (0.603), ε^2^ = 0.000	1.901 (0.386), ε^2^ = 0.000	1.669 (0.434), ε^2^ = 0.000	0.286 (0.867), W = 0.020	3.677 (0.159), W = 0.230	0.250 (0.882), W = 0.016
End Point Excursion	0.294 (0.863), ε^2^ = 0.000	0.324 (0.850), ε^2^ = 0.000	3.523 (0.172), ε^2^ = 0.076	3.298 (0.192), ε^2^ = 0.065	1.143 (0.565), W = 0.082	3.250 (0.197), W = 0.203	1.867 (0.393), W = 0.117
Maximum Excursion	0.643 (0.725), ε^2^ = 0.000	0.271 (0.873), ε^2^ = 0.000	2.996 (0.224), ε^2^ = 0.050	1.238 (0.538), ε^2^ = 0.000	6.231 (0.044), W = 0.445 *	0.600 (0.741), W = 0.037	1.867 (0.393), W = 0.117
Directional Control	0.378 (0.828), ε^2^ = 0.000	4.492 (0.106), ε^2^ = 0.125	1.442 (0.486), ε^2^ = 0.000	6.214 (0.045), ε^2^ = 0.211 *	6.000 (0.050), W = 0.429	4.710 (0.095), W = 0.294	12.250 (0.002), W = 0.766 *

Note. KW = Kruskal–Wallis; Δ = posttest–pretest. ε^2^ = epsilon-squared effect size for Kruskal–Wallis comparisons; Kendall’s W = Friedman effect size. * *p* < 0.05.

## Data Availability

The data presented in this study are available from the corresponding author upon reasonable request.

## Supplementary Materials

The following supporting information can be downloaded at: https://www.mdpi.com/article/10.3390/healthcare14183006/s1, Table S1: Completed CONSORT 2025 Checklist.

## Abbreviations

The following abbreviations are used in this manuscript:

CONSORT	Consolidated Standards of Reporting Trials
CTSIB	Clinical Test of Sensory Interaction on Balance
EG1	Once-weekly exercise group
EG2	Twice-weekly exercise group
LOS	Limits of Stability
SD	Standard deviation
SEM	Standard error of the mean

## References

[1] Horak F.B., Henry S.M., Shumway-Cook A. (1997). Postural perturbations: New insights for treatment of balance disorders. Phys. Ther..

[2] Fortin C., Ehrmann Feldman D., Cheriet F., Labelle H. (2011). Clinical methods for quantifying body segment posture: A literature review. Disabil. Rehabil..

[3] Julienne A., Verbecque E., Besnard S. (2024). Normative data for instrumented posturography: A systematic review and meta-analysis. Front. Hum. Neurosci..

[4] Lininger M.R., Leahy T.E., Haug E.C., Bowman T.G. (2018). Test-retest reliability of the Limits of Stability test performed by young adults using NeuroCom VSR Sport. Int. J. Sports Phys. Ther..

[5] Pickerill M.L., Harter R.A. (2011). Validity and reliability of limits-of-stability testing: A comparison of 2 postural stability evaluation devices. J. Athl. Train..

[6] Zemková E., Zapletaláková L. (2022). The role of neuromuscular control of postural and core stability in functional movement and athlete performance. Front Physiol..

[7] Summers S.J., Antcliff S., Waddington G., Wallwork S. (2022). Reliability and learning effects of repeated exposure to the Bertec Balance Advantage sensory organisation test in healthy individuals. Gait Posture.

[8] Muehlbauer T., Brueckner D., Schedler S. (2022). Effect of practice on learning a balance task in children, adolescents, and young adults. Front. Psychol..

[9] Grasso C., Barresi M., Tramonti Fantozzi M.P., Lazzerini F., Bruschini L., Berrettini S., Andre P., Dolciotti C., De Cicco V., De Cicco D. (2023). Effects of a short period of postural training on postural stability and vestibulospinal reflexes. PLoS ONE.

[10] Zhong Y., Chen P., Guo W., Wang X., Xue Y., Chen J., Liu J. (2025). Effects of different neuromuscular training modalities on balance performance in older adults: A systematic review and network meta-analysis. Front. Physiol..

[11] Silva A.G., Lã F.M.B., Afreixo V. (2015). Pain prevalence in instrumental musicians: A systematic review. Med. Probl. Perform. Artist..

[12] Baadjou V.A.E., Roussel N.A., Verbunt J.A.M.C.F., Smeets R.J.E.M., de Bie R.A. (2016). Systematic review: Risk factors for musculoskeletal disorders in musicians. Occup. Med..

[13] Kok L.M., Huisstede B.M.A., Voorn V.M.A., Schoones J.W., Nelissen R.G.H.H. (2016). The occurrence of musculoskeletal complaints among professional musicians: A systematic review. Int. Arch. Occup. Environ. Health.

[14] Kochem F.B., Silva J.G. (2018). Prevalence of playing-related musculoskeletal disorders in string players: A systematic review. J. Manip. Physiol. Ther..

[15] Rodríguez-Gude C., Taboada-Iglesias Y., Pino-Juste M. (2023). Musculoskeletal pain in musicians: Prevalence and risk factors—A systematic review. Int. J. Occup. Saf. Ergon..

[16] Cerda-Díaz L., Antúnez-Riveros M., Bracchiglione J., Opazo-Campusano R., Núñez-Cortés R. (2026). Musculoskeletal disorders in musicians: An overview of systematic reviews. Occup. Med..

[17] Kenny D.T., Ackermann B. (2015). Performance-related musculoskeletal pain, depression and music performance anxiety in professional orchestral musicians: A population study. Psychol. Music.

[18] Zão A., Altennmüller E., Azevedo L. (2025). Performance-related pain among professional orchestra musicians—The first Portuguese nationwide study (POMPS). Eur. J. Pain.

[19] Nusseck M., Spahn C. (2020). Comparison of postural stability and balance between musicians and non-musicians. Front. Psychol..

[20] Rousseau C., Barton G., Garden P., Baltzopoulos V. (2023). Assessing posture while playing in musicians—A systematic review. Appl. Ergon..

[21] Ajidahun A.T., Mudzi W., Myezwa H., Wood W.A. (2017). Musculoskeletal problems among string instrumentalists in South Africa. S. Afr. J. Physiother..

[22] Steinmetz A., Seidel W., Muche B. (2010). Impairment of postural stabilization systems in musicians with playing-related musculoskeletal disorders. J. Manip. Physiol. Ther..

[23] Blanco-Piñeiro P., Díaz-Pereira M.P., Martínez A. (2017). Musicians, postural quality and musculoskeletal health: A literature’s review. J. Bodyw. Mov. Ther..

[24] Chan C., Driscoll T., Ackermann B. (2014). Exercise DVD effect on musculoskeletal disorders in professional orchestral musicians. Occup. Med..

[25] Seidi F., Bayattork M., Minoonejad H., Andersen L.L., Page P. (2020). Comprehensive corrective exercise program improves alignment, muscle activation and movement pattern of men with upper crossed syndrome: Randomized controlled trial. Sci. Rep..

[26] Stanhope J., Pisaniello D., Weinstein P. (2022). The effect of strategies to prevent and manage musicians’ musculoskeletal symptoms: A systematic review. Arch. Environ. Occup. Health.

[27] Kiepe M.S., Fernholz I., Schmidt T., Brinkhaus B., Schmidt A., Weikert C., Rotter G. (2020). Effects of osteopathic manipulative treatment on musicians: A systematic review. Med. Probl. Perform. Artist..

[28] Laseur D.J.G., Baas D.C., Kok L.M. (2023). The prevention of musculoskeletal complaints in instrumental musicians: A systematic review. Med. Probl. Perform. Artist..

[29] Roos M., Lamontagne M.E., Desmeules F., Dionne C., Savard I., Pinard A.M., Lafrance S., Tanguay M., Roy J.-S. (2024). Workplace injury prevention and wellness program for orchestra musicians: A randomized controlled trial. J. Orthop. Sports Phys. Ther..

[30] Cherubini G., De Marco M., Converti R.M., Ramella M., Macchi C., Perucca L., Baccini M., Cecchi F. (2025). Impact of manual therapy on instrumentalist musicians with playing-related musculoskeletal disorders: A systematic review. Arch. Rehabil. Res. Clin. Transl..

[31] Eleryan N., Hemming R., Sparkes V., Sheeran L. (2025). Digital health self-management interventions for musicians with playing-related musculoskeletal disorders: A scoping review. Musculoskelet. Sci. Pract..

[32] Rotter G., Noeres K., Fernholz I., Willich S.N., Schmidt A., Berghofer A. (2020). Musculoskeletal disorders and complaints in professional musicians: A systematic review of prevalence, risk factors, and clinical treatment effects. Int. Arch. Occup. Environ. Health.

[33] Assel C., Nugraha B., Kallusky N., Faßnacht-Lenz S., Altenmüller E., Gutenbrunner C., Sturm C. (2023). Effect of manual therapy on music students with playing-related musculoskeletal disorders: A prospective randomized controlled trial. Front Pain Res..

[34] Baek C.Y., Ahn J.H., Lee J., Lee H.H., Lim W.T., Park H.K., Kim H.-D. (2025). Effect of digital health corrective posture exercise program on head and shoulder posture in adolescents: A cluster randomized controlled trial. Medicine.

[35] Acet N., Atalay Güzel N., Günendi Z. (2024). Effects of cervical mobilization on balance and proprioception in patients with nonspecific neck pain. J. Manip. Physiol. Ther..

[36] Zaidi S., Khan S.A., Zaki S., Sundus H., Alam M.F., Nuhmani S. (2025). Effectiveness of sensorimotor training on pain, cervical joint position sense, range of motion, balance, and disability in chronic neck pain: A randomized controlled trial. Heliyon.

[37] Chan C., Ackermann B. (2014). Evidence-informed physical therapy management of performance-related musculoskeletal disorders in musicians. Front. Psychol..

[38] Hopewell S., Chan A.W., Collins G.S., Hróbjartsson A., Moher D., Schulz K.F., Tunn R., Aggarwal R., Berkwits M., A Berlin J. (2025). CONSORT 2025 statement: Updated guideline for reporting randomised trials. BMJ.

[39] Hopewell S., Chan A.W., Collins G.S., Hróbjartsson A., Moher D., Schulz K.F., Tunn R., Aggarwal R., Berkwits M., A Berlin J. (2025). CONSORT 2025 explanation and elaboration: Updated guideline for reporting randomised trials. BMJ.

[40] Faul F., Erdfelder E., Lang A.G., Buchner A. (2007). G*Power 3: A flexible statistical power analysis program for the social, behavioral, and biomedical sciences. Behav. Res. Methods.

[41] Hoffmann T.C., Glasziou P.P., Boutron I., Milne R., Perera R., Moher D., Altman D.G., Barbour V., Macdonald H., Johnston M. (2014). Better reporting of interventions: Template for intervention description and replication (TIDieR) checklist and guide. BMJ.

[42] Virtanen P., Gommers R., Oliphant T.E., Haberland M., Reddy T., Cournapeau D., Burovski E., Peterson P., Weckesser W., Bright J. (2020). SciPy 1.0: Fundamental algorithms for scientific computing in Python. Nat. Methods.

[43] Lesinski M., Hortobágyi T., Muehlbauer T., Gollhofer A., Granacher U. (2015). Effects of balance training on balance performance in healthy older adults: A systematic review and meta-analysis. Sports Med..

[44] Gebel A., Lesinski M., Behm D.G., Granacher U. (2018). Effects and dose-response relationship of balance training on balance performance in youth: A systematic review and meta-analysis. Sports Med..

[45] Zalpour C., Ballenberger N., Avermann F. (2021). A physiotherapeutic approach to musicians’ health—Data from 614 patients from a physiotherapy clinic for musicians (INAP/O). Front. Psychol..

[46] Dijkman B., Kooistra B., Bhandari M. (2009). Evidence-Based Surgery Working Group. How to work with a subgroup analysis. Can. J. Surg..

