# Supplementary material for: Mobilization-Based Exercise Frequency and Dynamic Postural Control in Orchestra Musicians: An Exploratory Controlled Intervention Study
Source: Healthcare (Basel). 2026 Sep 14;14(18):3006. doi: 10.3390/healthcare14183006 (PMC13606296; doi:10.3390/healthcare14183006)
Supplement: Supplementary file 1 [file healthcare-14-03006-s001.zip › healthcare-4403327-supplementary.pdf]

Supplementary Table S1. Completed CONSORT 2025 Checklist

Manuscript: Mobilization-Based Exercise Frequency and Dynamic Postural Control in Orchestra Musicians: An Exploratory Controlled Intervention Study.

Trial registration: ClinicalTrials.gov Identifier NCT07632950 (retrospective registration; first submitted 2 June 2026, first posted 8 June 2026).

Note on scope: the allocation procedure could not be documented from contemporaneous records, so the study is reported as a non-randomized exploratory controlled intervention study. The CONSORT 2025 items are used below to structure transparent reporting; items that presuppose a documented randomized design are answered by stating what is and is not available rather than by asserting compliance.

Item	CONSORT 2025 requirement	Location in manuscript	Author response / status
1a	Title identifies the study as a randomized trial.	Title page	The title does not designate the study as a randomized trial. Because the allocation procedure cannot be evidenced, the title identifies the report as "An Exploratory Controlled Intervention Study".
1b	Structured summary of trial design, methods, results, and conclusions.	Abstract	Structured abstract reports design, groups, sample, outcomes, statistics, and conclusions, and states that the study is non-randomized and retrospectively registered.
2	Trial registry name, identifying number, URL, and date.	Abstract; Methods 2.1; Clinical Trial Registration statement	ClinicalTrials.gov Identifier: NCT07632950. Registration was retrospective: first submitted 2 June 2026 and first posted 8 June 2026, after the study was completed on 5 May 2026. Reported in Methods 2.1 and in the Clinical Trial Registration statement.
3	Where protocol and statistical analysis plan can be accessed.	Methods 2.1 and 2.5	No protocol or statistical analysis plan predating examination of the data exists or is publicly available; this is transparently disclosed rather than inferred.
4	Where and how deidentified participant data, code, and materials can be accessed.	Data Availability Statement	Data availability statement specifies availability from the corresponding author upon reasonable request, with confidentiality restrictions.
5a	Sources of funding/support and role of funder.	Funding; Conflicts of Interest	Funding source and funder role reported.
5b	Financial and other conflicts of interest.	Conflicts of Interest	Authors declare no conflicts of interest.
6	Scientific background and rationale.	Introduction	Rationale for musician postural-control demands and mobilization-based exercise reported.
7	Specific objectives related to benefits and harms.	Introduction	Aim and expectation reported; the outcome designation is explicitly described as post hoc. Harms monitored during sessions.
8	Patient/public involvement.	Methods / Checklist comment	Not applicable; no patient/public involvement in design, conduct, or reporting.
9	Trial design: type, allocation ratio, framework.	Methods 2.1 and 2.3	Three-group, repeated-measures, exploratory controlled intervention study; approximate 1:1:1 assignment ratio; not designated as a randomized trial because the allocation procedure cannot be evidenced.
10	Important changes to protocol after commencement.	Checklist comment	No protocol exists against which changes could be documented; no changes to the planned four-week intervention or the measurement schedule were reported by the investigators.
11	Trial setting and locations.	Methods 2.1 and 2.2	University orchestra musicians at Inonu University, Turkiye; single site.
12a	Eligibility criteria for participants.	Methods 2.2	Inclusion and exclusion criteria reported.
12b	Eligibility criteria for sites/intervention providers, if applicable.	Checklist comment	Not applicable; single-site supervised exercise intervention.
13	Intervention and comparator details sufficient for replication.	Methods 2.6	Methods 2.6 reports exercise content, sets/repetitions/hold times, supervised pain-free execution, 30-60 s rest/transition intervals, fixed volume, frequency dose, stopping criteria, session duration, adherence, and comparator (TIDieR).
14	Prespecified primary and secondary outcomes with time points.	Methods 2.5 and 2.7	No outcome was prespecified. LOS Reaction Time is reported as the primary outcome, but this designation was made during preparation of the statistical analysis, after data collection and inspection of the data; the retrospective registry entry names only a generic primary outcome ("change in postural control"). All other LOS and CTSIB variables are secondary and exploratory. Time points: baseline, Week 1, Week 4.
15	How harms were defined and assessed.	Methods 2.6; Results	Adverse events monitored each session; no adverse events reported.
16a	Sample size determination and assumptions.	Methods 2.4	Methods 2.4 reports G*Power version, f = 0.25 as a conventional medium planning effect because no pilot or directly comparable endpoint data were available, 80% power, alpha = .05, and the target sample.
16b	Interim analyses/stopping guidelines.	Methods 2.4	No interim analyses or stopping guidelines planned.
17a	Who generated allocation sequence and method used.	Methods 2.3	Not documented. No allocation list, randomization log, or protocol predating enrolment could be located, and the retrievable records do not identify the sequence-generation method or the responsible personnel. Methods 2.3 states this explicitly and the study is therefore not designated as randomized.
17b	Type of randomization/restrictions.	Methods 2.3	Not documented. Methods 2.3 reports approximate 1:1:1 assignment; no evidence of blocking, stratification, or any other restriction could be retrieved.

18	Allocation concealment mechanism.	Methods 2.1 and 2.3	Allocation concealment was not implemented.
19	Implementation and access to sequence.	Methods 2.3	Not documented. The personnel who implemented the assignment could not be identified from the retained records, and allocation concealment was not implemented.
20a	Who was blinded after assignment.	Methods 2.1 and 2.3; Limitations	No assessor blinding; disclosed as a limitation.
20b	How blinding was achieved/intervention similarity.	Methods; Limitations	Not applicable; blinding was not implemented and no sham comparator was used.
21a	Statistical methods for outcomes including harms.	Methods 2.7	Kruskal-Wallis, Mann-Whitney, Friedman, Wilcoxon, epsilon-squared, and Kendall’s W reported, with Bonferroni adjustment for post-hoc comparisons.
21b	Definition of analysis population.	Methods 2.7; Results	All allocated participants were analyzed in their assigned groups; no post-allocation exclusions.
21c	Handling of missing data.	Methods 2.7; Results	No missing outcome data; no imputation required.
21d	Additional analyses and whether prespecified/post hoc.	Methods 2.7	No subgroup or sensitivity analyses were performed. Because no protocol or statistical analysis plan predated the analysis, all reported analyses are post hoc in the strict sense and are presented as exploratory.
22a	Numbers randomly assigned, received intervention, and analyzed.	Figure 1; Results	Numbers allocated, treated, and analyzed are reported: Control n = 7, EG1 n = 8, EG2 n = 8; all analyzed. Figure 1 describes the flow as allocation rather than randomization.
22b	Losses/exclusions after randomization with reasons.	Figure 1; Results	No dropouts and no post-allocation exclusions.
23a	Recruitment/follow-up dates for outcomes and harms.	Methods 2.1; Results	The study ran from 5 March 2026 to 5 May 2026; follow-up was completed at Week 4. Ethics approval preceded enrolment (16 December 2024).
23b	Why trial ended/stopped, if relevant.	Methods 2.4; Checklist comment	The study ended after the planned four-week intervention; the target sample was limited by the accessible population.
24a	Intervention/comparator delivery, adherence, fidelity.	Methods 2.6; Results	Supervised sessions, 100% adherence, no adverse events.
24b	Concomitant care received by group.	Checklist comment	Not reported; no concomitant care formed part of the intervention protocol.
25	Baseline demographic and clinical characteristics by group.	Table 1	Age and sex distribution reported by group, with a note that the study was not powered to confirm baseline equivalence.
26	Numbers analyzed, available data, results, effect size/precision.	Tables 2-5; Results	n values, descriptive statistics, test statistics, p-values, and effect sizes reported; 95% CIs were not calculated for the non-parametric exploratory outcomes.
27	All harms/unintended events in each group.	Methods 2.6; Results	No adverse events reported.
28	Ancillary analyses.	Methods 2.7; Results	No ancillary subgroup or sensitivity analyses performed.
29	Interpretation consistent with results and harms.	Discussion	Interpretation is expressed as association and as a preliminary between-group statistical signal; no claim of efficacy or causality is made.
30	Limitations including bias, imprecision, generalizability, multiplicity.	Discussion limitations	Undocumented allocation procedure, retrospective registration, post hoc primary-outcome designation, small sample, lack of allocation concealment, blinding and sham comparator, practice effects, unmeasured confounders, no follow-up, and multiplicity are all reported.

Note. This checklist documents transparent reporting. Items marked as not documented, not available, or not applicable are disclosed rather than inferred. Entries revised in response to the editorial decision of 3 September 2026 are highlighted in yellow.
